# Contrasting Temporal Sequencing Effects of Self-Regulation, Mood, and Self-Efficacy Changes Within National Cancer Institute-Certified Obesity Intervention Processes Targeting Multiple Weight-Loss Behaviors in Community Settings

**DOI:** 10.3390/bs15121624

**Published:** 2025-11-25

**Authors:** James J. Annesi

**Affiliations:** Kinesiology Department, School of Health Sciences and Human Services, California State University, Monterey Bay, Seaside, CA 93955, USA; jamesannesi@gmail.com

**Keywords:** self-regulation, self-efficacy, mood, exercise, physical activity, eating, obesity, behavioral, treatment

## Abstract

For behavioral obesity treatments to improve their typically minimal effects and reduce an increasing reliance on surgical and pharmacologic interventions, an enhanced understanding of theory-driven psychosocial factors is needed. Recent applied research successfully targeted the social cognitive theory-related variables of self-efficacy (SE), self-regulation (SR), and mood (e.g., total mood disturbance; TMD) to increase participants’ exercise outputs and improve their dietary behaviors to sustain weight losses. However, an improved understanding of interactions of changes in those psychosocial factors, especially within paths toward longer-term behavioral changes, is required to increase both the reliability and effectiveness of obesity interventions. Women (*N* = 106) participated in a 12-month, community-based cognitive–behavioral obesity intervention. Consistent with tenets of social cognitive theory, most of the treatment time was focused on building SR and SE related to both exercise and controlled eating, and improving mood (i.e., TMD), primarily through increased exercise. Improvements in SR, SE, and TMD were significant (*p*s < 0.001) from baseline to Months 3, 6, and 9 (except in SE over 9 months). These effect sizes were large (*d*s = 0.82–1.53). In line with the prediction of both exercise and dietary improvements over 12 months, paths from 3-month, to 6-month, to 9-month changes in SR → TMD → SE (B = 0.93, *SE*_B_ = 0.59, 95% CI [0.034, 2.274], and B = 0.46, *SE*_B_ = 0.25, 95% CI [0.035, 1.025], respectively) and TMD → SR → SE (B = −0.08, *SE*_B_ = 0.03, 95% CI [−0.145, −0.020], and B = −0.04, *SE*_B_ = 0.01, 95% CI [−0.070, −0.015], respectively) were significant, whereas the other possible paths incorporating changes in SR, SE, and TMD were not. Consistent with social cognitive theory and the mood–behavior model, findings supported the value of early treatment foci on interactions of TMD and SR changes in pursuit of longer-term advancements in SE and weight-loss behaviors.

## 1. Introduction

In the United States (U.S.), the high prevalence of obesity (body mass index [BMI] ≥ 30 kg/m^2^) is of extreme concern. It presently affects 46% of women of ages 25 years and older and is expected to rise to ~60% by 2050 (GBD 2021 US Obesity Forecasting Collaborators, 2024). Obesity is consistent with health risks including type 2 diabetes mellitus, various cancers and heart diseases, kidney and liver diseases, depression, and sleep apnea (Bray et al., 2017). If left unabated, obesity rates will further challenge healthcare systems (Wolfenden et al., 2019). Problems with obesity prevalence are also increasing internationally as toxic food environments and sedentary lifestyles become more commonplace and difficult to counter (Blüher, 2019). On an individual level, although an improved diet (e.g., increased fruits/vegetables, decreased sweets; Dreher & Ford, 2020) along with increased physical activity/exercise is reliably associated with a decrease in excess weight, adherence to both of those behaviors has been *exceedingly* problematic beyond the short term (Dombrowski et al., 2014; MacLean et al., 2015). Although energy deficits are more pronouncedly related to dietary changes, improvements in both eating and exercise behaviors are required for sustained reductions in weight (Catenacci & Wyatt, 2007).

Possibly because of the considerable effort and general lack of success at controlling weight through behavioral means, use of pharmacologic (Berning et al., 2025) and surgical (Altieri et al., 2021) methods has increased. These methods have demonstrated reliable benefits for weight loss that include losses in excess in those who have demonstrated even the most productive behavioral approaches (Barrett et al., 2025). Behavioral researchers, however, suggest that better incorporation of health behavior change theory into treatment architectures can improve weight management effects, even without a reliance on surgeries or lifelong usages of medications (Baranowski et al., 2009; Gillison et al., 2015). Other researchers have advocated an approach that balances the pros and cons of medical and behavioral approaches (Tomiyama, 2025). Still others have suggested benefits of “stepwise approaches” that start with behavioral or lifestyle intervention (Kantowski et al., 2024). The large majority of behavioral weight-loss treatments rely on simply providing dietary and exercise information (Dombrowski et al., 2014; MacLean et al., 2015; Wadden et al., 2014). They mistakenly (and atheoretically) appear to expect participants’ inherent volition to sustain their required behavioral changes.

A predictive model proposed by Baker and Brownell (2000) linked changes in exercise, diet, and weight loss to psychological changes in self-efficacy (SE; feelings of ability), self-regulation (SR), mood, physical self-concept, and body image/body satisfaction. Subsequent intervention research evaluating their propositions suggested that the majority of the variance in changes in both eating and exercise behaviors (leading to weight loss) was explained by treatment-associated changes in only SE, SR, and overall mood (Annesi & Johnson, 2015). Each of these factors have been supported in the treatment-associated reduction in weight (SE, Burke et al., 2015; SR, Teixeira et al., 2015; mood; Blaine et al., 2007). When evaluated together, 6-month changes in each of those three variables explained a significant, unique portion of the variance in improved exercise and fruit/vegetable intake leading to weight loss (Annesi, 2012). This validated the importance of addressing SE, SR, and overall mood in behavioral weight-loss intervention processes. Fruit/vegetable intake has been shown to be an indicant of the overall health of an individual’s diet (Aljadani et al., 2013), especially when sweet consumption was also accounted (Annesi, 2016).

The above findings were useful in informing the foci of novel cognitive–behavioral weight-loss interventions (NIH/National Cancer Institute, 2025). However, *when* each psychological factor should be emphasized within a treatment protocol targeting both exercise and dietary behaviors remained unclear. It was also deemed important to assess interrelationships between changes in SE, SR, and mood across time frames leading to the behavioral predictors of weight loss, especially that maintained beyond an initial 6–9 months (where weight regains typically occur; Dombrowski et al., 2014; MacLean et al., 2015). Suggestions were that analyses of possible paths toward behavioral changes should be driven, wherever possible, by theory, especially where multiple behaviors (e.g., exercise and eating) are the targets of change (Noar et al., 2008; Sheeran et al., 2017). For example, the mood–behavior model (Gendolla, 2000) suggests that initial mood improvement empowers extended advancements in SR that are associated with positive changes in behaviors—especially goal-directed behaviors (i.e., improved mood → increased SR → improved weight management behaviors). Social cognitive theory (Bandura, 1986, 2000) and self-regulation theory (Vohs & Baumeister, 2017) intimate that early increases in SR skill usage empowers subsequent improvements in mood and SE, as what had been perceived as exceedingly challenging behaviors improve. This facilitates an enhanced psychological atmosphere and increased feelings of ability which lend themselves to sustained improvements in behavior (i.e., improved SR → improved mood → improved weight management behaviors; improved SR → increased SE → improved weight management behaviors). Self-efficacy theory (Bandura, 1997), rather, suggests that early changes in SE facilitates longer-term improvements in mood and energizes internal resources such as in one’s self-management to persevere though personal barriers (i.e., increased SE → improved mood → increased SR → improved weight management behaviors). Coaction theory (Johnson et al., 2014) posits that changes in one health behavior carry over to other health behaviors through psychological mechanisms that include SE and SR. Thus, a unified measure of both SE and SR—here across exercise and dietary contexts—would be justified. By establishing temporal sequencing of changes in SE, SR, and mood in the predictions of exercise and dietary changes, theory, research, and obesity treatment contents and applications might be meaningfully refined.

Women, more than men, report attempts at weight loss through behavioral (Zhong et al., 2022), pharmacologic (Börchers & Skibicka, 2025), and surgical (Young et al., 2016) means. Because psychological variables have greater effects on the weight management efforts of women (Smith et al., 2020), research addressing those variables should account for the sexes separately (Zhong et al., 2022). Also, it has been suggested that there are sex-specific differences in responses to dietary, behavioral, and pharmacological interventions, and even in treatment-related adverse events (Kantowski et al., 2024). Thus, within this investigation, only women with obesity enrolled in a community-based weight management program were included. It was expected that there would be significant within-group improvements in the targeted psychological variables of SE, SR, and overall negative mood (total mood disturbance; TMD)—and in exercise and dietary behaviors—associated with a cognitive–behavioral obesity program. It was also predicted that improvements in exercise and diet would be significantly associated with lost weight. However, because behavioral theories suggested differing temporal sequencing of changes in the assessed psychosocial variables in the predictions of both exercise and dietary changes, expressions of significance of paths incorporating their progressive changes (e.g., change over 3 months predicting changes over 6 months, and so on) was left as a research question, without a hypothesis. To contribute to treatment contents that target both exercise and eating changes simultaneously, of primary interest here was identification of a path (paths) that significantly predicted improvements in *both* exercise and diet.

## 2. Materials and Methods

### 2.1. Participants

Participants were part of an ongoing initiative of field-based research contrasting behavioral weight management methods within U.S. community wellness settings (Annesi, 2022). They responded to information about volunteering provided through local newspapers and social media. For the present investigation, data from the most recent group of women (minimum age of 21 years) with obesity prior to the disruption associated with the COVID-19 pandemic, and also fulfilling the additional inclusion criteria, were incorporated. The additional inclusion criteria were (a) physical condition enabling safe participation; (b) no current/soon-planned pregnancy; and (c) within the previous 12 months, no weight management program participation or start/change in one or more prescribed psychoactive medication. The self-reported weight/height ratio consistent with obesity was cross-checked by study staff. There was no cost or compensation for participating. The sample size (*N* = 106) approximated that required for the planned regression analyses annotated in the below Data Analyses Subsection. At baseline, the age range was 21–59 years (*M* = 46.6 years, *SD* = 9.6), the BMI range was 30.0–40.7 kg/m^2^ (*M* = 34.5 kg/m^2^, *SD* = 3.2), and the racial/ethnic make-up was 70% White, 23% Black, and 7% Hispanic. The educational levels were 74% bachelor’s degree or greater and 26% high school or college. Nearly all participants were within a middle yearly family income range of USD 50,000–USD 150,000, with a median reported family income of USD 72,500/year. Ethical requirements of the World Medical Association Declaration of Helsinki and the American Psychological Association were maintained. A university institutional review board approved the study protocol which required signed informed consent by each participant.

### 2.2. Measures

SE, within this study, was regarded as the respondent’s degree of confidence and perceived ability at persisting with the weight-loss behaviors of exercise and controlled eating under challenging conditions. It was measured as an aggregate of SE for exercise through use of the 5-item Exercise Self-Efficacy Scale (e.g., “I feel I don’t have the time”; Marcus et al., 1992), and SE for controlled eating through use of the 20-item Weight Efficacy Life-Style Questionnaire (e.g., “I can resist eating even when others are pressuring me to eat”; Clark et al., 1991). After a minor adjustment to obtain symmetry across the scales’ original scoring systems, possible response ranges were 1 (*not confident*) to 10 (*very confident*). Participants’ mean response scores were calculated by scale and weighted for the number of items in each scale. Thus, the possible score range was 1–10, with a higher value indicating more SE. Internal consistencies for the Exercise Self-Efficacy Scale were reported at Cronbach’s α = 0.76–0.82, and test–retest reliabilities over 2 weeks were 0.74–0.78 (Marcus et al., 1992). Internal consistencies for the Weight Efficacy Life-Style Questionnaire were reported at Cronbach’s α = 0.76–0.82 (Clark et al., 1991). For the present sample, the correlation between baseline scores on SE for exercise and SE for controlled eating was *r* = 0.58, *p* < 0.001, and the Cronbach’s α on the aggregated SE scale was 0.77.

SR was considered to be the degree the respondent presently uses self-management methods/skills to deal with challenges and barriers to exercise and controlled eating. It was measured by aggregating item responses from the 10-item Exercise-Related Self-Regulation Scale (e.g., “I say positive things to myself about being physically active”; Annesi & Marti, 2011) and the 10-item Eating-Related Self-Regulation Scale (e.g., “I make formal agreements with myself regarding my eating”; Annesi & Marti, 2011). The possible response range for each scale was 1 (*never*) to 4 (*often*). Participants’ mean response scores were calculated by scale and weighted for the number of items in each scale. Thus, the possible score range was 1–4, with a higher value indicating more use of SR methods. Internal consistencies were reported at Cronbach’s α = 0.79 and 0.81, respectively. Test–retest reliabilities over 2 weeks were reported at 0.78 and 0.74, respectively (Annesi & Marti, 2011). For the present sample, the correlation between baseline scores on the Exercise-Related Self-Regulation Scale and Eating-Related Self-Regulation Scale was *r* = 0.64, *p* < 0.001, and the Cronbach’s α on the aggregated SR scale was 0.77.

The 30-item TMD scale of the Profile of Mood States-Brief measured overall negative mood (McNair & Heuchert, 2009). Its six aggregated subscales of 5 items each were tension/anxiety (“nervous”), depression/dejection (“sad”), fatigue/inertia (“weary”), anger/hostility (“annoyed”), confusion/bewilderment (“confused”), and vigor/activity (“energetic”). Possible response ranges were 0 (*not at all*) to 4 (*extremely*) for each item, and they were summed after following the developers’ directions to add a constant of +4 to the confusion/bewilderment subscale score total and reverse-score the items related to vigor/activity. Thus, the possible score range of TMD was −16 to 104, with a higher value indicating a more negative mood (thus, a lower score is more favorable). Internal consistencies were reported at Cronbach’s α = 0.76–0.92, with the present sample ranging from α = 0.80–0.91. Test–retest reliabilities over 3 weeks were reported at 0.65–0.74 (McNair & Heuchert, 2009).

Exercise outputs were measured by the Godin–Shephard Leisure-Time Physical Activity Questionnaire (Godin, 2011). It also assessed physical activity, which is considered to be a less-structured form of exercise. The number of exercise sessions of “mild intensity” (e.g., normal-paced walking), “moderate intensity” (e.g., fast-paced walking), and “strenuous intensity” (e.g., running) with a duration of at least 15 min during the previous 7 days were recalled by the respondent. Each of those sessions was accorded a score of 3, 5, or 9 metabolic equivalents (METs), respectively. A MET is a measure of energy expenditure beyond one’s resting state. After multiplying the number of sessions by its MET value for each entry, they were summed. A higher score indicated more completed exercise. Godin–Shephard Leisure-Time Physical Activity Questionnaire scores demonstrated compelling evidence of validity through its relationships with accelerometry, body fat, and VO_2_ max treadmill test results, with 2-week test–retest reliability reported at 0.74 (Amireault & Godin, 2015; Amireault et al., 2015; Jacobs et al., 1993; Miller et al., 1994; Pereira et al., 1997). The Godin–Shephard Leisure-Time Physical Activity Questionnaire has been incorporated frequently in medical research (Sikes et al., 2019).

Overall dietary behavior was quantified using a previously applied method of aggregating recalled food intake (Annesi, 2016). Scoring weights the positive effects of combined fruit and vegetable intake (×2) minus the detrimental effects of sweets (×1) (Aljadani et al., 2013; Drewnowski et al., 2004; Te Morenga et al., 2012). Portion sizes and corresponding instructions (that included adjustments for small/large portions, mixed foods [e.g., salads], and omissions of fried fruits/vegetables) were based on U.S. government sources (U.S. Department of Agriculture, 2017; U.S. Department of Health and Human Services and U.S. Department of Agriculture, 2015). Examples were a moderate-size (118 mL) pear, moderate serving (118 mL) of green beans, a small (59 mL) piece of cake, and a cup (237 mL) of full-sugar soda. When a fruit/vegetable was combined with a sweet (e.g., caramel-coated apple), it was to be counted as a sweet. Instructions required the participant’s recall of her consumptions during “a typical day over the previous 7 days.” A higher score indicated a healthier diet. The 3-week test–retest reliabilities in women were reported at 0.77–0.83, and concurrent validity was demonstrated through correspondences with well-validated food recall measures requiring lengthier administration times (e.g., Block Food Frequency Questionnaire; Block et al., 1986; Mares-Perlman et al., 1993).

The weight of participants was measured to the nearest 0.10 kg using a medical-grade digital scale (Health O Meter 80 KL; McCook, IL, USA). It was re-calibrated the day of each measurement. Each participant complied with a request to remove her shoes and any heavy outer clothing such as a jacket prior to being measured. To confirm the participants’ required criterion of having obesity (BMI ≥ 30 kg/m^2^) at baseline, height was also measured at that time to the nearest 0.10 cm using a stadiometer (Health O Meter Portrod; McCook, IL, USA).

### 2.3. Procedure

Treatment instructors were existing staff members of the participating community wellness centers. Each of those instructors indicated a desire to be involved in the research. They were trained by study staff in the 14-month treatment protocol, requiring a total of ~25 h from each participant. The treatment contents were adapted from a program certified by the Research-Tested Intervention Programs and the Evidence-Based Cancer Control Programs of the National Institutes of Health/National Cancer Institute of the U.S. (NIH/National Cancer Institute, 2025). The intervention protocol was guided primarily by tenets of social cognitive theory (Bandura, 1986), self-regulation theory (Vohs & Baumeister, 2017), self-efficacy theory (Bandura, 1997), and the mood–behavior model (Gendolla, 2000). Each of these paradigms emphasize the potential for goals such as increased exercise, improved dietary behaviors, and weight loss to be positively affected by attention to environmental, psychosocial, and behavioral factors, as well as the over-time interactions of their changes. Thus, there was an emphasis within the treatment on increasing SR and SE to induce improvements in weight-loss behaviors and on increasing exercise outputs to improve mood and its additional behavioral effects (Arent et al., 2020), as well as provide some energy-expenditure and metabolic benefits (Donnelly et al., 2009). There was a combination of 45 to 50 min individual and small-group instruction sessions, generally convening every 2 weeks after several weekly meetings initially.

Guided by self-regulation theory (Vohs & Baumeister, 2017), self-management methods previously proposed for improving multiple health behaviors (Michie et al., 2011) were addressed. The SR skills development of the participants included instruction and rehearsals in goal setting/progress tracking, relapse prevention, cognitive restructuring, stimulus control, and attention control (e.g., dissociation from discomfort). Consistent with social cognitive theory and self-efficacy theory (Bandura, 1986, 1997; Waddington, 2023), increases in SE were sought through the suggested processes of (a) “mastery experience” (by emphasizing that one’s SR skill usage helps to successfully manage challenges); (b) “social persuasion” (by instructor-supplied verbal encouragement); (c) “vicarious reinforcement” (by instructor-provided examples of successes from participants’ peers); and (d) “emotional states” (through improvements in mood fostering a positive internal atmosphere). The association of enhanced mood with behavioral improvements suggested by the mood–behavior model (Gendolla, 2000) was bolstered through instructor-led training in breath control and abbreviated progressive muscle relaxation (Muhammad Khir et al., 2024). Consistent with coaction theory (Johnson et al., 2014), generalization of both SR and SE across exercise and controlled eating behaviors was sought throughout.

In terms of specific behavioral foci within the treatment, while the suggested ≥ 150 min/week of moderate or greater intensity exercise for health benefits (U.S. Department of Health and Human Services/Office of Disease Prevention and Health Promotion, 2023) was mentioned, simply increasing cardiovascular exercise using participant-preferred modalities was the treatment-enacted goal. Treatment-directed goals for dietary behaviors emphasized increasing fruit/vegetable intake, minimizing sweets, and maintaining a daily energy intake of 1200–1500 kilocalories/day. These directives were based on research indicating that only three sessions of moderate exercise/week are associated with improved mood and weight in adults with obesity (Annesi, 2012), and an increase in fruit/vegetable intake positively affects other food groups, the overall health of the diet, and body composition (Aljadani et al., 2013; Annesi, 2016). Because of research indicating its benefits (Zheng et al., 2015), weekly self-weighing was promoted to participants by instructors. Because of high degrees of intervention time placed on behavior-change methods, participants were directed to the U.S. government website, myplate.gov, for additional education when/if desired.

In-person fidelity checks structured by a form requiring study staff ratings of each instructor’s adherence to treatment-related processes within the assigned time frames indicated strong protocol compliance. The same study staff who conducted fidelity assessments also administered study measures to participants in a private area where the associated data were kept anonymous and confidential.

### 2.4. Data Analyses

There was no systematic bias (White et al., 2011) found in the presence/absence of the 13% of missing data, all beyond baseline. This missing-at-random status fulfilled the condition required for application of the expectation–maximization algorithm for imputation (Little & Rubin, 2014). For the primary regression analyses, a sample size of ≥98 was required to detect the moderate effect of *f*^2^ = 0.15 at the conservative statistical power of 0.90, α < 0.05 (Cohen et al., 2003). Variance inflation factor values of <2.0 indicated no issues with multicollinearity, with no observed floor or ceiling effects.

A series of *t* tests separately assessed significance of within-group gains (changes) in SE, SR, and TMD from baseline to Months 3, 6, and 9, and changes in exercise output and the overall diet from baseline to Month 12. To enable assessment of temporal sequencing of changes in the psychosocial variables in the predictions of exercise and dietary changes, a series of path analyses was conducted. Because this study focused on multiple weight-loss behavior change processes, primary attention was on paths that significantly predicted *both* exercise and dietary changes. The entire possible matrix of temporally ordered changes (Δ) in psychosocial predictors (Δbaseline–Month 3 → Δbaseline–Month → 6 → Δbaseline–Month 9) to Δbaseline–Month 12, in both behavioral variables, were separately assessed. These included changes in SE → SR → TMD, SR → TMD → SE, SR → SE → TMD, TMD → SR → SE, SE → TMD → SR, and TMD → SE → SR. Consistent with suggestions (Hayes et al., 2017), this method enabled evaluation of the significance of designated sets of temporally ordered relationships among psychosocial variables leading to the behavioral changes of interest over 12 months. Finally, a sensitivity analysis assessed the association of weight change over 12 months with exercise and dietary changes (simultaneously entered into a multiple regression equation).

Statistical testing was facilitated by SPSS Statistics Version 28.0 along with the PROCESS 4.2 macroinstruction Model 6, incorporating 10,000 percentile-based bootstrapped resamples (Hayes, 2022). Statistical significance was set at α < 0.05 (two-tailed). Where bootstrapping was incorporated, a 95% confidence interval (95% CI) was used to determine statistical significance. Due to possessing theoretical bases, and consistent with suggestions for similar analyses (Francis & Thunell, 2021; Perneger, 1998), there was no adjustment of significance level for the multiple tests.

## 3. Results

Table 1 displays descriptive data of the psychosocial and behavioral variables at the assessed temporal points, along with their change terms. Changes over each period indicated significant improvements associated with the treatment (*p*s < 0.001), with large effect sizes (*d*s > 0.80). The only exception was with a reduction in SE when assessed from baseline to Month 9. A follow-up analysis suggested that this was associated with a significant decline in participants’ SE between Month 6 and Month 9.

Table 2 presents path data on the matrix of temporally ordered psychosocial changes’ effect on exercise and dietary behaviors. The only significant paths to both exercise and dietary improvement were from SR → TMD → SE (B = 0.93, *SE*_B_ = 0.59, 95% CI [0.034, 2.274] and B = 0.46, *SE*_B_ = 0.25, 95% CI [0.035, 1.025], respectively) and TMD → SR → SE (B = −0.08, *SE*_B_ = 0.03, 95% CI [−0.145, −0.020] and B = −0.04, *SE*_B_ = 0.01, 95% CI [−0.070, −0.015], respectively). None of these four significant paths demonstrated a significant direct effect between the independent and outcome (dependent) variables (*p*s > 0.05). Both of the significant paths initiated from the interaction of SR and TMD changes during the initial 6 months. Although of minimal interest based on the aims of this study, two other paths toward only exercise were significant. They were SR → SE → TMD (B = 2.61, *SE*_B_ = 0.89, 95% CI [1.224, 4.586]) and TMD → SE → SR (B = −0.15, *SE*_B_ = 0.05, 95% CI [−0.271, −0.070]).

Within the sensitivity analysis, participants’ weight loss from baseline (*M* = 94.86 kg, *SD* = 11.59) to Month 12 (*M* = 89.32 kg, *SD* = 11.95) was significant, *t*(105) = 12.59, *p* < 0.001, 95% CI [4.67, 6.41], *d* = 1.22. This represented a mean weight loss of 5.8% (−5.54 kg) which was significantly associated with the exercise and dietary changes, *R* = 0.30, *R*^2^ = 0.09, *F*(2,103) = 5.25, *p* = 0.007, η^2^ = 0.09.

## 4. Discussion

Consistent with the aims of this field-based investigation, findings contributed to obesity treatment theory and provided material that could aid in future treatment applications. This was accomplished through assessment of the matrix of six possible paths of sequential changes over 3, 6, and 9 months in SE, SR, and TMD resulting in 12-month changes in exercise, dietary behaviors, and weight. Congruent with coaction theory (Johnson et al., 2014), these psychosocial factors were deemed to be interrelated across exercise and eating contexts and, thus, measured in an aggregate manner. As recently suggested (Chao et al., 2025), the results of the present female sample also built upon, and extended, previous research that demonstrated salience in behavioral obesity treatments focused on fostering improvements in theory-driven psychosocial factors over simply increasing participants’ knowledge about diet and exercise (still the prevailing method).

In support of the initial hypothesis and previous research that used more static analytic formats, the cognitive–behavioral treatment was associated with significant improvements in the targeted psychosocial variables of SE, SR, and TMD, as well as in exercise and the overall diet. Also, as expected, the improvements in diet and exercise significantly predicted weight loss. The mean weight reduction of −5.8% over 12 months suggested that there were meaningful declines in health risks in the great majority of participants (Williamson et al., 2015). Their body composition improvements were associated with exercise increases from a mean of (the equivalent of) ~1.5 moderate–vigorous exercise sessions/week to ~6.5 sessions/week (beyond the minimum government recommendation; U.S. Department of Health and Human Services/Office of Disease Prevention and Health Promotion, 2023). Also, their average daily fruit/vegetable intake (a primary indicant of the overall health of a diet; Aljadani et al., 2013; Drewnowski et al., 2004) approximately doubled to surpass the recommended five daily portions (Rooney et al., 2017).

Regarding the research question and central inquiry within this study, findings supported the value of early treatment attention placed on interactions of mood and SR changes in the quest for longer-term improvements in SE. Consistent with social cognitive theory, self-efficacy theory (Bandura, 1986, 1997), and self-regulation theory (Vohs & Baumeister, 2017), the increases in SE over 9 months were significantly associated with improvements in both exercise and dietary behaviors, inducing weight loss beyond the expected periods of regain after 6–9 months (Dombrowski et al., 2014; MacLean et al., 2015). Although improvements in SR, SE, and mood had previously been identified as key predictors of weight-loss behavior improvements (Annesi, 2012; Baker & Brownell, 2000; Blaine et al., 2007; Burke et al., 2015; Teixeira et al., 2015), neither their interactions nor sequential effects were addressed in a comprehensive enough manner to inform treatment architectures to the level presented here. In many cases, evaluations were over the short term using cross-sectional analyses and did not account sufficiently for the most central shortcoming in behavioral obesity treatments—regain of weight after several months of loss (Blomain et al., 2013; Dombrowski et al., 2014; MacLean et al., 2015). Thus, several refinements in theory-to-practice application were enabled here by addressing those limitations and gaps. Also, because improvements in SR, SE, and mood were previously represented as each having a *direct* relationship with either behavioral changes or weight (Blaine et al., 2007; Burke et al., 2015; Teixeira et al., 2015), interventionists might have been led to focus on only one of these psychosocial changes.

While the paths addressed in this report occur whether they are measured or not, the enhanced clarity given the present analyses allows behavioral interventionists to both address multiple (overlapping) psychological variables and do so in an informed manner that prioritizes their attention within a treatment timeline. For example, the present results suggest that if adherence to exercise is treated as an initial focus and supported by attention to SR skills development, exercise-related mood improvements will interact with the enhanced self-management abilities and also enable an SR focus on dietary behaviors (Johnson et al., 2014; Oaten & Cheng, 2006). Following this, these psychosocial improvements will continue to reinforce SE, which has been identified within both theory and research as empowering the required sustenance of behavioral changes required for long-term weight loss (Burke et al., 2015; Holloway & Watson, 2002). Even given promising findings in the behavioral treatment domain, more research will be needed on the comparative effects of improved behavioral approaches to weight loss vs. pharmacological and surgical approaches. Additionally, more investigation into conditions where combined approaches might be a basis for additional benefits for the many adults with obesity is needed.

### Limitations

Even given its contributions, limitations were present within this study. For example, in extensions of this research, incorporation of control and/or contrast groups will help account for possible confounding related to volunteerism, expectation, and social support effects. In this domain, accounting for the effects of the social networks of participants on obesity treatment effects might be particularly salient (Christakis & Fowler, 2007; Gesell et al., 2013). Also, the sample was homogeneous. Thus, assessment of generalizability of findings is needed through evaluations of men; adults of specific ethnic/racial, socioeconomic, and age groups; and individuals with medical issues beyond obesity. Randomized control designs will also improve the strength of findings. Better accounting for scores related to dropout or participation rate might also prove useful. Additionally, the present research had a high dependence on (validated) self-report measures—requiring accurate behavioral recalls from participants. Although the selection of psychosocial variables for testing was based on both theory and research, other theoretical models might suggest other factors of value for future related inquiry. Consideration of how bidirectional relationships among the variables affect findings should also be given more attention in replications of this research. Finally, with larger sample sizes and greater experimental power, the use of structural equation modeling might serve to extend the present form of analyses.

## 5. Conclusions

A paradigm for multiple simultaneous health–behavior changes should carefully address their sequential interactions on a psychosocial predictor level, as occurred here. Theoretically driven intervention models that draw from such findings represent an advancement and will lead to increased success in treating obesity through behavioral means (or as an adjunct to medical interventions). When accomplished within field settings, as within this investigation, theory can be leveraged to induce needed behavioral changes to many individuals in community environments that are highly accessible. For example, the present findings suggest interventions should allocate initial sessions to SR skills and mood enhancement via exercise. Continuity with related research remains necessary to further refine behavioral treatment contents and processes and weigh their strengths and shortcomings against pharmacological and surgical approaches in adults with obesity. Such findings could have national policy implications as countries seek to temper obesity-related issues and associated medical expenditures.

## Figures and Tables

**Table 1:** Descriptive data and gain scores on the assessed psychological and behavioral variables (*N* = 106).

Baseline			Month 3		Month 6		Month 9		Month 12	
			[ΔBaseline-Month 3]		[ΔBaseline-Month 6]		[ΔBaseline-Month 9]		[ΔBaseline-Month 12]	
	*M*	*SD*	*M*	*SD*	*M*	*SD*	*M*	*SD*	*M*	*SD*
Self-Efficacy	4.78	1.50	6.17	1.56	6.57	1.59	4.08	1.28		
			[1.39]	[1.69]	[1.79]	[1.78]	[−0.70]	[1.65]		
Self-Regulation	1.86	0.43	2.55	0.32	2.62	0.30	2.56	0.31		
			[0.69]	[0.53]	[0.76]	[0.50]	[0.70]	[0.53]		
Total Mood Disturbance	25.70	15.36	10.77	14.62	5.67	11.66	7.98	11.83		
			[−14.92]	[14.05]	[−20.02]	[17.28]	[−17.72]	[17.15]		
Exercise (METs/week)	8.52	7.26							32.66	15.32
									[24.14]	[15.29]
Overall Diet	5.72	4.42							11.11	4.61
									[5.39]	[5.35]

Δ—change during the designated temporal period (given within brackets).

**Table 2:** Relationships within paths toward changes in exercise and diet (*N* = 106).

ΔBaseline- Month 3	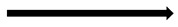	ΔBaseline- Month 6	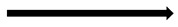	ΔBaseline- Month 9	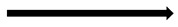	ΔBaseline- Month 12
	B (*SE*_B_) [95% CI]		B (*SE*_B_) [95% CI]		B (*SE*_B_) [95% CI]	
Self-Efficacy	0.15 (0.02) [0.101, 0.200]	Self-Regulation	−6.11 (3.55) [−13.155, 0.939]	Mood Disturb.	−0.20 (0.08) [−0.360, −0.042]	Exercise
	0.15 (0.02) [0.101, 0.200]		−6.11 (3.55) [−13.155, 0.939]		−0.05 (0.03) [−0.110, 0.010]	Diet
**Self-Regulation**	**−6.90 (3.21) [−13.277, −0.530]**	**Mood Disturb.**	**−0.03 (0.01) [−0.049, −0.021]**	**Self-Efficacy**	**3.87 (0.98) [1.941, 8.810]**	**Exercise**
	**−6.90 (3.21) [−13.277, −0.530]**		**−0.03 (0.01) [−0.049, −0.021]**		**1.92 (0.35) [1.226, 2.609]**	**Diet**
Self-Regulation	2.16 (0.25) [1.665, 2.661]	Self-Efficacy	−5.91 (1.09) [−8.064, −3.753]	Mood Disturb.	−0.20 (0.08) [−0.364, −0.045]	Exercise
	2.16 (0.25) [1.665, 2.661]		−5.91 (1.09) [−8.064, −3.753]		−0.04 (0.03) [−0.097, 0.023]	Diet
**Mood Disturb.**	**−0.02 (0.00) [−0.022, −0.010]**	**Self-Regulation**	**1.26 (0.29) [0.692, 1.834]**	**Self-Efficacy**	**3.69 (0.92) [1.862, 5.512]**	**Exercise**
	**−0.02 (0.00) [−0.022, −0.010]**		**1.26 (0.29) [0.692, 1.834]**		**1.96 (0.34) [1.291, 2.621]**	**Diet**
Self-Efficacy	−4.46 (0.93) [−6.307, −2.608]	Mood Disturb.	−0.002 (0.00) [−0.007, 0.003]	Self-Regulation	13.70 (2.76) [8.219, 19.172]	Exercise
	−4.46 (0.93) [−6.307, −2.608]		−0.002 (0.00) [−0.007, 0.003]		2.78 (1.04) [0.719, 4.850]	Diet
Mood Disturb.	−0.07 (0.01) [−0.091, −0.051]	Self-Efficacy	0.20 (0.03) [0.150, 0.256]	Self-Regulation	10.48 (2.97) [4.587, 16.366]	Exercise
	−0.07 (0.01) [−0.091, −0.051]		0.20 (0.03) [0.150, 0.256]		1.86 (1.13) [−0.386, 4.100]	Diet

Disturb.—Disturbance. Δ—change during the designated temporal period. Arrows denote directionality within the evaluated paths. Mood Disturbance—Total Mood Disturbance scale of Profile of Mood States. B—beta value. *SE*_B_—standard error associated with the beta value. 95% CI—95% confidence interval. B, *SE*_B_, and 95% CI intervals were derived from 10,000 bootstrapped resamples of the data. Data from significant paths toward changes in both exercise and diet are given in bold.

## Data Availability

Based on institutional review stipulations for participant anonymity and privacy, the data set supporting this study’s findings will be made available by reasonable request made to the corresponding author.

## Abbreviations

SR—self-regulation; SE—self-efficacy; TMD—total mood disturbance.
